# MiRNA199a-3p suppresses tumor growth, migration, invasion and angiogenesis in hepatocellular carcinoma by targeting VEGFA, VEGFR1, VEGFR2, HGF and MMP2

**DOI:** 10.1038/cddis.2017.123

**Published:** 2017-03-30

**Authors:** Alip Ghosh, Debanjali Dasgupta, Amit Ghosh, Shrabasti Roychoudhury, Dhiraj Kumar, Mahadeo Gorain, Ramesh Butti, Simanti Datta, Shaleen Agarwal, Subash Gupta, Gopal Krishna Dhali, Abhijit Chowdhury, Thomas D Schmittgen, Gopal C Kundu, Soma Banerjee

**Affiliations:** 1 Center for Liver Research, School of Digestive and Liver Diseases, Institute of Post Graduate Medical Education and Research, Kolkata, India; 2 Laboratory of Tumor Biology, Angiogenesis and Nanomedicine Research, National Centre for Cell Science (NCCS), Pune 411007, India; 3 Center for Liver and Biliary Sciences, Indraprastha Apollo Hospital, New Delhi, India; 4 Division of Gastroenterology, School of Digestive and Liver Diseases, Institute of Post Graduate Medical Education and Research, Kolkata, India; 5 Department of Hepatology, School of Digestive and Liver Diseases, Institute of Post Graduate Medical Education and Research, Kolkata, India; 6 College of Pharmacy, University of Florida, Gainesville, FL, USA

## Abstract

Increasing significance of tumor–stromal interaction in development and progression of cancer implies that signaling molecules in the tumor microenvironment (TME) might be the effective therapeutic targets for hepatocellular carcinoma (HCC). Here, the role of microRNA miR-199a-3p in the regulation of TME and development of HCC has been investigated by several *in vitro* and *in vivo* assays. Expression of miR-199a-3p was observed significantly low in HCC tissues and its overexpression remarkably inhibited *in vivo* tumor growth and metastasis to lung in NOD-SCID mice. *In vitro* restoration of miR-199a-3p expression either in endothelial cells (ECs) or in cancer cells (CACs) significantly diminished migration of ECs in co-culture assay. Again incubation of miR-199a-3p transfected ECs with either conditioned media (CM) of CACs or recombinant VEGF has reduced tube formation, in ECs and it was also dropped upon growth in CM of either anti-VEGF antibody-treated or miR-199a-3p-transfected CACs. In addition, bioinformatics and luciferase-reporter assays revealed that miR-199a-3p inhibited VEGF secretion from CACs and VEGFR1 and VEGFR2 expression on ECs and thus restricted cross talk between CACs and ECs. Again, restoration of miR-199a-3p in hepatic stellate cells (HSCs) reduced migration and invasion of CACs in co-culture assay, while it was enhanced by the overexpression of HGF suggesting miR-199a-3p has hindered HSC-CACs cross talk probably by inhibiting HGF and regulating matrix metalloproteinase MMP2, which were found as targets of miR-199a-3p subsequently by luciferase-reporter assay and gelatin zymography, respectively. Thus, these findings collectively highlight that miR-199a-3p restricts metastasis, invasion and angiogenesis in HCC and hence it may be considered as one of the powerful effective therapeutics for management of HCC patients.

Reciprocal signaling between tumor cells and the stromal components of surrounding tumor microenvironment (TME) is the fundamental to the evolution and metastasis of solid tumors including hepatocellular carcinoma.^1, 2, 3, 4^ This complex dynamic network orchestrated mainly by cancer cells (CACs) and coherently activated stromal cells (SCs) such as fibroblasts or cancer-associated fibroblasts (CAFs), hepatic stellate cells (HSCs), endothelial cells (ECs; tumor-associated ECs), non-hepatic tumor infiltrating immune cells.^5^ In addition to extracellular matrix (ECM) proteins, myriads of chemokines, cytokines and soluble growth factors are indispensible to the cross talk between CACs and TME.^6^ Under normal physiological microenvironment, tissue integrity is maintained by intercellular adhesive interactions that control cellular proliferation, homeostasis and locomotion;^7^ but in cancer, TME experiences drastic changes, which supports uncontrolled proliferation, resisting cell death, inducing angiogenesis, activating invasion and metastasis through the cross talk between CACs and SCs.^4^

During HCC progression in response to paracrine signal from injured hepatocytes, normal fibroblasts or HSCs differentiate into myofibroblast-like cells,^8, 9^ which then secrete many mitogenic and motogenic factors such as hepatocyte growth factor (HGF), fibroblast growth factor (FGF), platelet derived growth factor (PDGF), transforming growth factor *β*1 (TGF*β*1), matrix metalloproteinases (MMPs) and ECM to create a complex milieu in TME, whereas CACs exude TGF*β*1, PDGF, VEGF that activate other SCs.^10, 11, 12, 13^ Again, tumor-associated ECs stimulate HSCs to secrete VEGF and PDGF.^14^ VEGF functions through membrane-bound receptor tyrosine kinases VEGFR1/FLT1 and VEGFR2/KDR/Flk1 for the activation of kinase-dependent signaling cascades required for angiogenesis or blood vessel formation^15, 16^ through which CACs survive and also disseminate into circulation and extravasate to distal tissue or organs. However, the functional implication of this complex cross communications among CACs, HSCs, ECs and ECM remodeling, which is an important area of potential systematic drug development for the management of HCC, has not been explored explicitly.

MicroRNAs (miRNAs) are small endogenous noncoding RNA molecules with high post-transcriptional regulatory activity emerging as important driver in several aspects of tumor pathogenesis^17, 18, 19^ and also in different complex interactions in TME.^20, 21^ Recent evidences suggested that coordinated control of many genes by single miRNA is an ingenious mechanism to control disease pathogenesis.^22^ One such tumor suppressor miRNA is miR-199a-3p, which is silenced in many cancers such as ovarian, colorectal, renal, endometrial and HCC.^23, 24, 25, 26, 27, 28^ Reduced expression of miR-199a-3p is anticipated with the overexpression of HGF receptor cMET,^25^ the cell surface adhesion molecule CD44^26^ and mTOR of AKT/mTOR signaling pathway.^27^ In addition, we have observed in this study that restoration of miR-199a-3p restricted tumor growth and extrahepatic metastasis in mice and angiogenesis, migration and invasion of cancer cells by downregulating five new components from TME of HCC and thus abrogated cross talk between HSCs–CACs and CACs–ECs implicating high therapeutic potential of this miRNA for the management of HCC.

## Results

### miR-199a-3p is downregulated in HCC tissues

**Figure 1:**
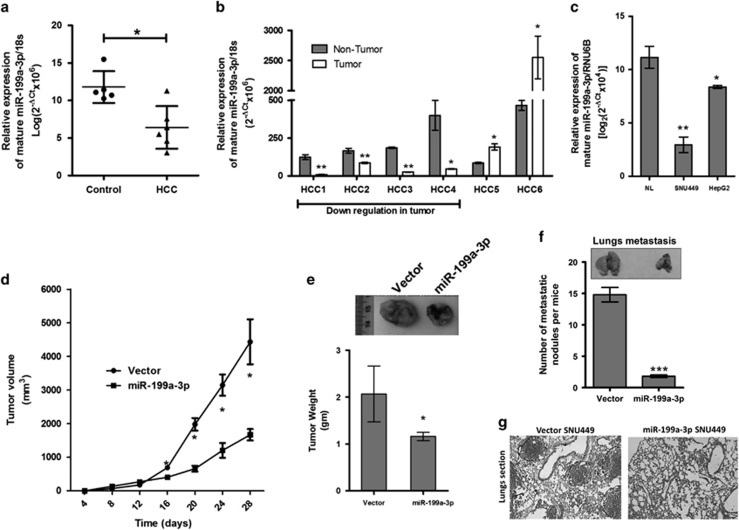
Anti-tumorigenic role of miR-199a-3p. miR-199a-3p expression was compared between (**a**) HCC tumor and normal liver (NL) tissues, (**b**) HCC tumor and adjacent non-tumor tissues and (**c**) HCC cell lines and normal liver (NL). (**d**) Tumor volume at different time points and (**e**) tumor weight of subcutaneous HCC in either vector or miR-199a-3p overexpressed cell line injected NOD/SCID mice. (**f**) Size and number of the nodule formed in the lung after 4 weeks of injection of either vector or miR-199a-3p overexpressed cell line through tail vein in NOD/SCID mice. (**g**) Hematoxylin and eosin staining of the lung section. Area enclosed by dotted line indicates metastatic growth. *P<0.05, **P<0.01 and ***P<0.005 respectively

The relative abundance of the mature miR-199a-3p in Indian HCC patients (*n*=6) was determined and compared with that of either adjacent non-tumor tissue or control liver tissue (*n*=5; Figures 1a and b). In accordance to the previous reports, a significant downregulation of this miRNA was observed in HCC tissues. In addition, its level was found significantly low in the HCC cell lines such as HepG2 and SNU449 compared with that of the average of five control liver tissues (Figure 1c).

### *In vivo* restoration of miR-199a-3p shows anti-tumorigenic activity associated with reduced angiogenesis

To examine the anti-tumorigenic role of miR-199a-3p, premiR-199a-3p overexpressing SNU449 cells (Supplementary Figure S1) were injected subcutaneously (s.c.) into the right flank of NOD/SCID mice. Tumor volume was measured twice a week upto 4 weeks. At the end of the experiments, the mice were killed; tumors were excised, weighed and photographed. Tumor growth was suppressed in the presence of miR-199a-3p as compared with vector control (Figures 1d and e). As the prognosis of HCC patients with extrahepatic metastasis remains poor and pulmonary metastasis is the chief site of spread, premiR-199a-3p overexpressing SNU449 cells were also injected through the lateral tail vein of female NOD/SCID mice, killed after 4 weeks of injection and metastatic colonies were counted in the lung section. The size and number of colonies in the lung was significantly low in these mice compared with vector cells injected mice as observed in the hematoxylin- and eosin-stained lung section (Figures 1f and g).

### miR-199a-3p inhibits tumor angiogenesis and migration by attenuating cross talk between CACs and ECs

**Figure 2:**
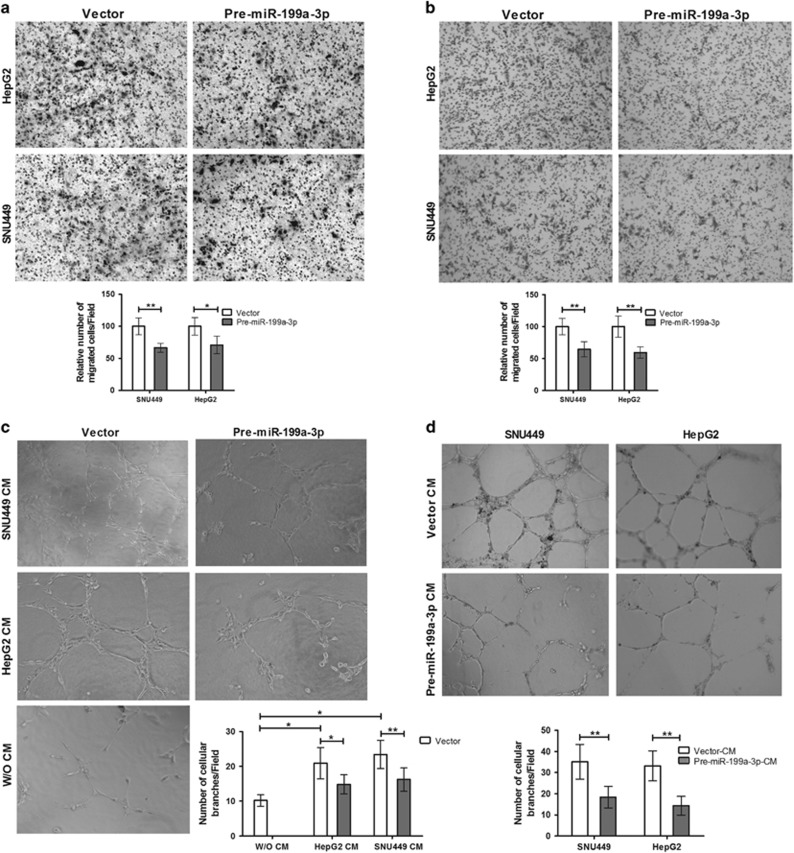
miR-199a-3p inhibits migration and angiogenesis in ECs by regulating cross talk between CACs and ECs. Endothelial cell recruitment assay in Boyden chamber: HUVEC cells were co-cultured with HepG2 and SNU449 separately. premiR-199a-3p and control vector plasmids were transfected either in (**a**) HUVEC or (**b**) in HCC cell lines. Bar diagram represented percentage of migrated cells though 8*μ*m filter. *In vitro* tube formation in ECs was performed after similar transfection in either (**c**) HUVEC or (**d**) HCC cell lines. HUVECs were cultured on matrigel-coated plate with CM of cancer cell lines. Bars represented percentage of cellular branches. All the experiments were repeated three times. *P<0.05 and **P<0.01, respectively

Angiogenesis is indispensible for cancer cell growth, migration, invasion and metastasis. VEGF–VEGFR signaling is the key EC specific signaling pathway required for angiogenesis and tumor vasculogenesis. To elucidate the effect of miR-199a-3p on HCC angiogenesis, *in vitro* endothelial recruitment and tube-formation assays were performed. HepG2, SNU449 and HUVEC cell lines were used to describe the paradigm of cross talk between transformed hepatocyte and ECs, respectively. In endothelial recruitment assay, HUVEC cells were transfected with either premiR-199a-3p plasmid or control vector and seeded on upper compartment of Boyden chamber and co-cultured with HCC cells (HepG2 and SNU449 independently) grown in the lower compartment. After 24 h, a significant reduction was noticed in the ability of migration of ECs to the lower surface of the membrane. Similarly, lower number of ECs was migrated on restoration of miR-199a-3p expression within HCC cells in co-culture condition (Figures 2a and b).

Again, compared with control vector-transfected HUVEC cells, significantly small number of capillary-like structures were developed on the matrigel on restoration of miR-199a-3p in HUVEC cells cultured in the presence of CM from two different HCC cell lines HepG2 (29%) and SNU449 (31%) separately as measured by the number of new cellular branches protruding from ECs (Figure 2c). Similarly, tube formation was reduced dramatically in HUVECs that were grown in the presence of CM from premiR-199a-3p-transfected HepG2 (57%) or SNU449 (48%) cells than control vector (Figure 2d). Thus miR-199a-3p inhibits invasion, migration and angiogenesis by reducing genes on both CACs and ECs and hence disrupts communication between them.

### miR-199a-3p inhibits angiogenesis by directly reducing VEGF secretion from CACs and suppressing expression of its receptors VEGFR1 and VEGFR2 on ECs

**Figure 3:**
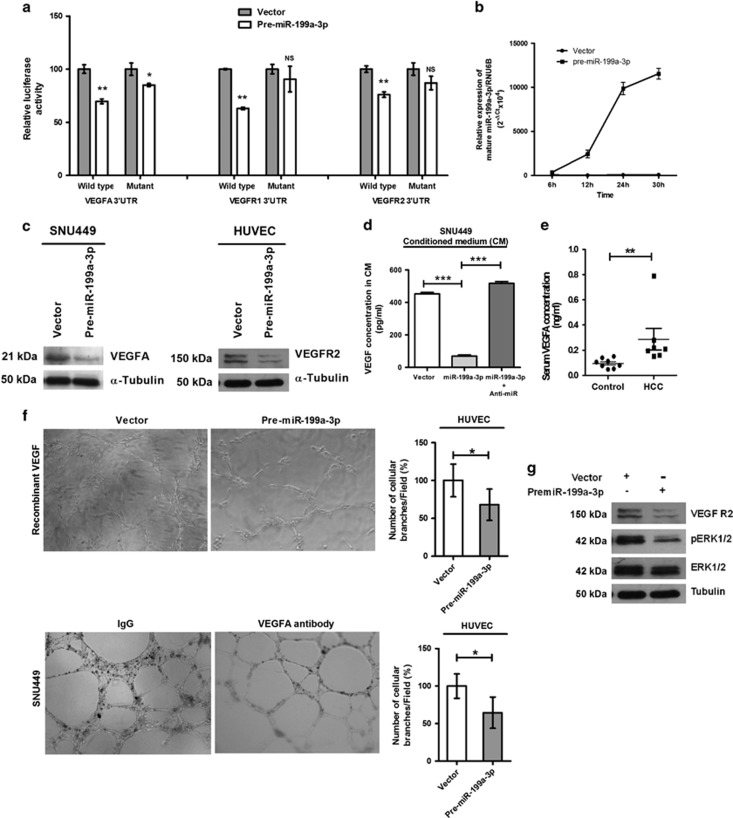
VEGFA in cancer cells and VEGFR1 and VEGFR2 on endothelial cells are suppressed by miR-199a-3p. (**a**) Luciferase-reporter assay with VEGFR2 were determined separately in premiR-199a-3p-transfected wild-type and miR-199a-3p binding-site mutant 3′-UTRs of VEGFA, VEGFR1, HepG2 cells. (**b**) Ectopic expression of miR-199a-3p was confirmed at different time points. (**c**) Protein expression of VEGFA and VEGFR2 in SNU449 and HUVEC cells, respectively. By ELISA, secreted VEGFA was determined in (**d**) culture media of control vector, premiR-199a-3p and premiR-199a-3p with anti-miR-199a-3p-transfected SNU449 cells and (**e**) serum of healthy volunteer (*n*=8) and HCC patients (*n*=7). (**f**) Tube-formation assay was performed with premiR-199a-3p or vector-transfected HUVECs in the presence of 20 ng/ml of recombinant VEGFA and anti-VEGF antibody or control IgG-treated CM of SNU449. (**g**) VEGFR2, ERK1/2 and phospho-ERK1/2 levels were determined in transfected HUVEC cells cultured in the presence of 20 ng/ml of recombinant VEGFA. *α*-Tubulin was used as loading control in western blot. *P<0.05, **P<0.01 and ***P<0.005

To understand the molecular mechanism of inhibition of angiogenesis, we used a systemic approach to identify molecular targets of miR-199a-3p. Using two different target prediction algorithms such as miRanda (microrna.org) and targetscan (targetscan.org), we found the complementary sequences of miR-199a-3p in the 3′-UTR of four important genes of angiogenesis VEGFA, VEGFR1, VEGFR2 and ANGPT1 (Supplementary Table 1). Therefore, we have selected these genes for further validation by *in vitro* experiments. Dual reporter luciferase assay revealed that the co-transfection of premiR-199a-3p and 3′-UTR construct of each gene significantly reduced the luciferase activities of VEGFA (30%), VEGFR1 (39%), VEGFR2 (24%) and ANGPT1 (39% Figure 3a and Supplementary Figure S2). VEGF–VEGFR signaling genes were further validated with miRNA binding-site mutants of miR-199a-3p (Figure 3a). Ectopic expression of mature miR-199a-3p at different time points was confirmed after transfecting the premiR-199a-3p plasmid in HepG2 cells by qRT-PCR (Figure 3b). Then the mRNA expression of VEGFA, VEGFR1 and VEGFR2 in HCC (HepG2 and SNU449) and HUVEC cell lines were determined to confirm the origin of expression of each protein (Supplementary Figure S3). The gain of function analysis of mir-199a-3p in respective cell lines had further confirmed that this miRNA diminished the expression of intracellular VEGFA in SNU449 and VEGFR2 in HUVEC as observed by western blot analysis (Figure 3c). Extracellular VEGFA level in the media of HCC cell line was also reduced in the presence of miR-199a-3p and it was reverted back to the normal level when treated with anti-miR-199a-3p as observed by ELISA (Figure 3d). High level of VEGFA was also noted in the serum of HCC patients with venous invasion (*n*=7) compared with healthy volunteers (*n*=8) (Figure 3e).

Furthermore, a dramatically reduced level of angiogenesis was noted either on overexpression of miR-199a-3p in ECs in the presence of recombinant VEGFA or addition of anti-VEGFA antibody-treated CM of SNU449 on HUVEC (Figure 3f) implicating that miR-199a-3p attenuated expression of VEGFR2 on ECs.

Thus these findings suggest that miR-199a-3p may repress migration and angiogenesis of ECs by inhibiting the secretion of VEGFA from HCC cells and subsequently abrogating the pro-angiogenic signaling pathway by targeting VEGFR2 on ECs.

### miR-199a-3p also blocks downstream signaling pathways in ECs

VEGFA secreted from CACs binds to VEGFR2 on ECs, phosphorylates and activates VEGFR2, which subsequently phosphorylates downstream extracellular signal-regulated kinase (ERK1/2) to promote angiogenesis. Compared with control vector, miR-199a-3p-transfected HUVEC cells incubated with recombinant VEGFA displayed relatively lower levels of both VEGFR2 and phosphorylated ERK1/2 (Figure 3g). Thus restoration of miR-199a-3p in ECs might suppress neoangiogenesis by inhibiting downstream angiogenic signaling pathways.

### Hepatocyte-stellate cells cross talk inhibited by miR-199a-3p attenuating invasion and metastasis of CACs

**Figure 4:**
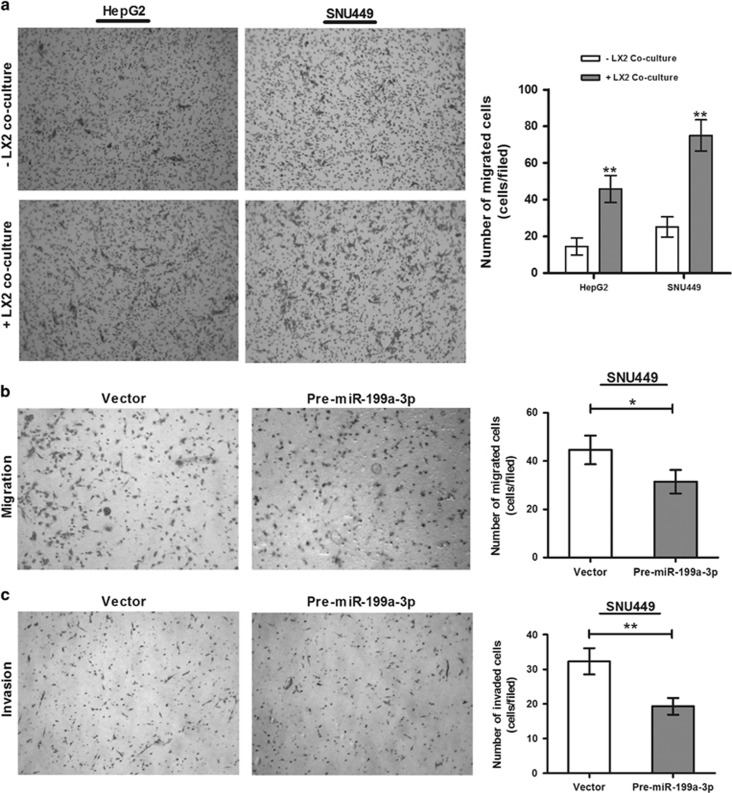
miR-199a-3p inhibits migration and invasion of cancer cells by regulating the cross talk between cancer cells (HepG2 or SNU449) and hepatic stellate cells (LX2). (**a**) Migrated cancer cells in transwell plate upon co-culturing with either LX2 cells or mock media. LX2 cells were transfected with vector and premiR-199a-3p, and SNU449 cells were either (**b**) co-cultured with transfected LX2 or (**c**) grown on matrigel pre-coated plate with CM of transfected LX2 cells. Average number of SNU449 cells migrated and invaded was counted. *P<0.05 and **P<0.01

To elucidate the role of miR-199a-3p in HCC metastasis, the effect of this miRNA on migration and invasion ability of CACs were investigated. Co-culturing of HCC cell lines (SNU449 and HepG2 separately) in the upper compartment of Boyden chamber with human stellate cells (LX2) in lower chamber revealed a dramatically higher level of migration of HCC cells compared with mock media (Figure 4a). In contrast, migration of CACs (SNU449) was drastically reduced on restoration of miR-199a-3p in LX2 cells (71%) compared with vector (100%) in the similar assay (Figure 4b).

Again, the invasion ability of HCC cells (SNU449) through matrigel was also declined significantly on incubation with CM of miR-199a-3p-transfected LX2 cells (60%) compared with vector (100% Figure 4c). These evidences strongly suggested that miR-199a-3p represses the cross talk between HSCs and CACs and thus inhibits invasion and migration of CACs.

### miR-199a-3p exerts its effect by negatively regulating HGF signaling

**Figure 5:**
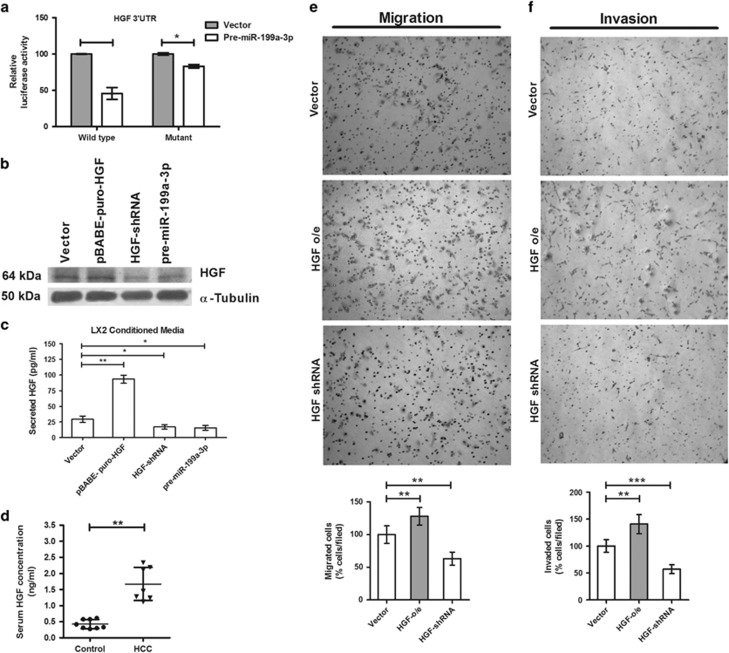
miR-199a-3p reduces secretion of HGF from LX2 cell line to inhibit its paracrine effect on cancer cells. (**a**) 3′-UTR-reporter-luciferase assay of HGF in HepG2 cells transfected with vector and premiR-199a-3p. LX2 cells were transfected with vector, pBABE-puro HGF, HGF-shRNA and premiR-199a-3p. Intracellular HGF expression was determined by (**b**) western blot and secreted HGF by ELISA in (**c**) cell culture media and (**d**) serum of HCC patients. (**e**) Migration and (**f**) invasion ability of SNU449 cells in the presence or absence of HGF secretion from LX2 was determined. *P<0.05, **P<0.01 and ***P<0.005

To understand the molecular mechanism by which miR-199a-3p inhibited tumor metastasis, one of the most attractive metastatic genes, hepatocyte growth factor (HGF) identified in bioinformatics analysis as its' target (Supplementary Table 1) was validated *in vitro*. Luciferase reporter assay with full-length 3′-UTR construct consisting of three miR-199a-3p binding sites revealed that the co-transfection of miR-199a-3p significantly reduced the luciferase activity to 45%, which was recovered to 83% with single miRNA binding-site mutant construct where two other binding sites were unaltered. Immunoblotting and confocal microscopy analysis in the presence or absence of miR-199a-3p consistently depicted that this miRNA directly regulated the expression of HGF (Figures 5a, b and Supplementary Figure S4). Inhibition of the intracellular protein expression of HGF by miR-199a-3p also reduced the secretion of HGF in the culture media of LX2 cells as confirmed by ELISA (Figure 5c). Moreover, blockage of HGF expression by shRNA mimics the effect of miR-199a-3p whereas overexpression of HGF was considered as positive control in western blot, confocal analysis and ELISA (Figures 5a, b and Supplementary Figure S4). The level of HGF was quantified significantly high in the serum of HCC patients as determined by ELISA (Figure 5d).

HGF originated from mesenchymal cells acts as a paracrine effector molecule for hepatocytes that express the receptor tyrosine kinase MET. Both gain or loss of migration and invasion ability of HCC cells in the presence of either HGF-overexpressed CM or HGF-depleted CM of LX2 confirmed that HGF is the metastatic signaling molecule in the cross talk between HSC and CACs (Figures 5e and f). In 2010, Fornari *et al.*^28^ had shown that miR-199a-3p inhibits HGF/cMET signaling by targeting MET in HCC. Thus, reduced migration and invasion ability of HCC cells (SNU449) when co-cultured with premiR-199a-3p-transfected HSCs (LX2) revealed that miR-199a-3p could diminish both HGF and cMET and thus abrogate the generation of signal in HSCs and its transmission into CACs.

### MMP2 signaling is also abrogated by miR-199a-3p

**Figure 6:**
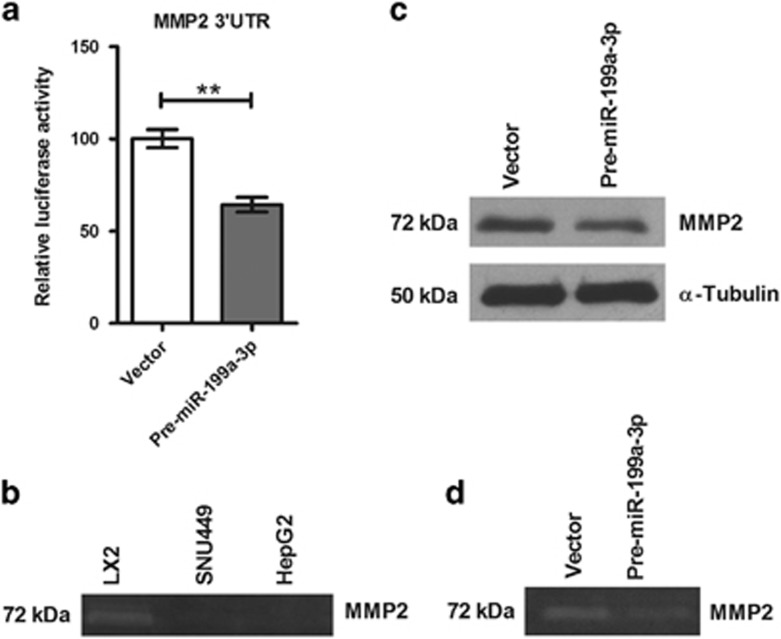
miR-199a-3p regulates extracellular matrix remodeling by targeting MMP2. (**a**) 3′-UTR-reporter-luciferase assay of MMP2 in HepG2 cells co-transfected with control vector and premiR-199a-3p. (**b**) MMP2 activity was determined by gelatin zymography in LX2, SNU449 and HepG2. (**c**) Western blot and (**d**) gelatin zymography of LX2 cells either transfected with premiR-199a-3p or control vector. **P<0.01

We further explored the molecular mechanisms of multifaceted role exerted by miR-199a-3p. Using bioinformatics analysis, MMP2 was also identified as a potential target of this miRNA (Supplementary Table 1). We also validated this target by *in vitro* assays as this gene is usually overexpressed in HCC and it is one of the crucial factors in metastasis and angiogenesis.^29^ Dual reporter luciferase assay with 3′-UTR construct showed that overexpression of miR-199a-3p significantly reduced the luciferase activity (Figure 6a). Gelatinolytic activity of MMP2 determined by gelatin zymography with CACs (SNU449 and HepG2) and HSCs extract revealed that MMP2 was secreted mostly by HSCs (Figure 6b). Moreover, the introduction of miR-199a-3p diminished the expression of MMP2 as observed in western blot analysis and its activity in gelatin zymography suggesting that miR-199a-3p exerts its effect by binding to the 3′-UTR of MMP2 gene (Figures 6c and d).

Collectively, our data signify that miR-199a-3p is a potent multifaceted tumor suppressor miRNA, which attenuates intercellular communications between HSCs–CACs and CACs–ECs, thereby inhibiting signal initiation, migration, metastasis and angiogenesis in HCC.

## Discussion

Complex dynamic interplay of malignant epithelial cells, multiple stromal cells and non-cellular components of surrounding TME are prerequisite for HCC onset and progression.^6^ However, the underlying molecular mechanisms involved in this cross talk are poorly understood. Recent reports suggest that inflammation and activation of HSCs in a bidirectional cross talk between CACs and HSCs orchestrate a favorable microenvironment for the growth of HCC^6, 7^ and TGF*β* mediates cross talk between malignant hepatocyte and tumor-associated macrophages (TAM) through ECM remodeling and angiogenesis.^30^ We further demonstrated here that a combined cross talk between CACs–ECs and CACs-activated HSCs through soluble mediators have indispensible role in HCC progression. In addition to the elucidation of the mechanism of HCC development, a single small noncoding multipotent tumor suppressor miRNA, miR-199a-3p, has been identified in this study using both *in vitro* and *in vivo* assays, which has robust potential to regulate multiple proteins involved in the intercellular signaling network executed during progression of liver diseases to HCC. *In vitro* co-culturing of each combination of cells such as CACs (HepG2 and SNU449)–ECs (HUVECs) and CACs–HSCs (LX2) as well as suppression of *in vivo* tumor growth and metastasis to lung in nude mice revealed that this miRNA regulated multiple aspects of HCC such as angiogenesis, invasion and metastasis by targeting three important pathways VEGF–VEGFR signaling, HGF–cMET signaling and ECM remodeling. Thus, by favorably modulating specific regulators of intercellular signaling pathways and components of TME, miR-199a-3p offers fascinating therapeutic potential for HCC.

HCC always develops in the background of fibrotic or inflamed tissue associated with distorted parenchyma, progressive capillarization and deposition of ECM, which create an intricate microenvironment. Therefore, a complex tumor–stromal interaction network persists from the very early stage of HCC development to provide a favorable environment to CACs to survive in the hypoxic condition, which requires angiogenesis to supply oxygen and nutrient. Recent evidences imply that miRNAs might be a new category of intercellular molecules that are emerging as of primary importance in controlling cross talk between CACs and components of TME.^22, 31, 32^ Using a mouse xenograft model, miR-126 and miR-126* have been identified as suppressor of sequential recruitment of mesenchymal stem cells and inflammatory monocytes to the tumor stroma to inhibit metastasis of breast cancer by targeting SDF1*α* in CACs.^31^ Overexpression of miR-101 in HCC mice model reduces epithelial–mesenchymal transition (EMT) and angiogenesis by repressing EZH2, COX2, STMN1 and ROCK2^22^ and miR-195 suppresses angiogenesis by reducing VEGF, VAV2 and CDC42.^32^ The tumor suppressor miRNA, miR-199a-3p is downregulated in HCC liver tissues as observed in several studies and its decrement significantly correlates with poor prognosis and survival of the patients, whereas restoration of miR-199a-3p in HCC cell line leads to reduced invasiveness and enhanced doxorubicin sensitivity by abrogating mTOR, CD44, and cMET proto-oncogene.^27, 28^ Reduced level of this miRNA was also observed in HCC liver tissues included in this study and restoration of this miRNA in CACs, ECs and HSCs was found to disrupt intercellular cross talk involved in angiogenesis and metastasis.

Five new effector genes of this miRNA has been identified and validated in this study. All of these genes were components of TME of HCC, which are either soluble growth factors or their receptors on cancer cells or stromal cells entailed in the angiogenic and metastatic signaling pathways. Among these genes, miR-199a-3p downregulated mostly the pro-angiogenic factor VEGFA, which is secreted from CACs and its receptors VEGFR1, VEGFR2 located on ECs. Co-culturing of ECs with HCC cells pre-transfected with premiR-199a-3p significantly reduced the migration ability of ECs in transwell assay, whereas restoration of miR-199a-3p expression in ECs suppressed the tube-formation ability in the presence of VEGF growth factor. These findings suggested that miR-199a-3p inhibited VEGF secretion from CACs and also attenuated its receptor-mediated downstream signaling via VEGFR2. Although VEGF and VEGFRs have been considered as therapeutic targets for inhibiting angiogenesis,^33, 34^ now it is apparent that targeting a single factor might not be adequate for cancer therapy. VEGF in combination with another factor, which is overexpressed in HCC, angiopoitin 2 (ANGPT2) functions synergistically in vascular remodeling during angiogenesis,^35^ whereas angiopoitin 1 (ANGPT1) is involved in the maturation and stabilization of newly formed blood vessels. In this study, ANGPT1 has been identified as a target of miR-199a-3p in bioinformatics analysis and 3′-UTR luciferase-reporter assay. But its synergistic effect on angiogenesis is not clear yet. Recently, Zhu *et al.*^36^ have reported that miR-146a upregulates PDGFR*α*, located on ECs, through upregulation of BRCA1 and enhances HCC progression by increasing microvascular invasion that lead to high HCC recurrence and poor survival. Bioinformatics analysis and luciferase-reporter assay indicated that the receptor of PDGF, PDGFR*α*, located on HSCs and ECs was another important target of miR-199a-3p (data not shown). Thus miR-199a-3p targets VEGFR1, VEGFR2 and probably PDGFR*α* on ECs and soluble factor VEGFA and ANGPT1 secreted from CACs to inhibit angiogenesis.

Although angiogenesis is essential for tumor growth and metastasis, in order to initiate vascular branching, MMPs are required to breach ECM, which facilitates the release of ECM-bound growth factors (VEGFA, FGF etc.) and helps in the migration of ECs.^37^ Overexpression of MMP2 has been observed in different cancers including HCC.^38^ The 3′-UTR of MMP2 has one miR-199a-3p binding site, which was verified with luciferase assay, western blot and zymography in this study. Thus, the reduced level of migration of ECs either transfected with premiR-199a-3p or incubated with the CM of premiR-199a-3p-transfected HCC cells could be due to synergistic effect of both VEGFA and MMP2.

More than 80% of liver cancer develops in the background of fibrotic liver implicating the role of activated HSCs in the pathogenesis of HCC. Using a co-culture model with activated HSCs and HCC cell lines, this study demonstrated that the cross talk between HSCs and CACs has great impact on migration and invasion of HCC cells. Activated HSCs secrete a broad range of extracellular proteins including HGF, and aberrant HGF/cMET signaling has a critical role in bidirectional cross talk between HSCs and CACs, which promotes invasion, migration and angiogenesis in HCC.^39, 40^ An inverse relation in cMET and miR-199a-3p has been reported in HCC recently.^28^ In our study, HGF was identified as a potent target of miR-199a-3p and co-culturing of HCC cells with premiR-199a-3p-transfected HSCs dramatically attenuated migration and invasion of HCC cells through matrigel by abrogating the cross talk between HSCs and CACs.

Recently the signaling cascades regulating the cross talk between HSCs–CACs and CACs–ECs have received much attention for drug target. Sorafenib is the only FDA-approved oral multi-tyrosine kinase receptor inhibitor of VEGFR2/3 and PDGFR recommended for the treatment of advanced HCC, which reduces growth of tumor by disrupting tumor–stromal interaction.^41^ It also attenuates fibrosis by reducing proliferation of HSCs and ECM deposition.^42^ However, Sorafenib has shown to prolong the median survival of HCC patients by only 3–4 months.^43, 44^ Thus miR-199a-3p investigated in this study, which attenuates multiple intercellular cross talks in HCC by abrogating several components of TME such as VEGFA, VEGFR1, VEGFR2, ANGPT1, MMP2, HGF, cMET, CD44 and PDGFR*α* might prove a valuable therapeutic molecule for HCC.

Given that TME has a pivotal role in HCC onset and progression, a promising anticancer therapeutic should simultaneously regulate multiple intercellular signaling pathways by aiming various components of it. Thus interfering appropriately with various routes of cross talk of CACs and stromal components of TME, mir-199a-3p has opened a new therapeutic avenue for the effective treatment of HCC patients. In addition, silencing of multiple signaling molecules of the liver microenvironment by this miRNA suggests that with improved understanding of the molecular mechanism of its function, this miRNA can also be used as therapeutic for management of early liver disease patients in the near future.

## Materials and methods

### Cell lines and culture conditions

Human HCC cell line HepG2 and SNU449 were cultured in Dulbecco's modified Eagle's medium (DMEM) and RPMI1640 from Life Technologies (Carlsbad, CA, USA) supplemented with 10% fetal bovine serum (Gibco, Grand Island, NY, USA), respectively. The Hepatic stellate cell line, LX2 was a kind gift from Professor Scott Friedman, Mount Sinai School of Medicine, USA and cultured in DMEM supplemented with 5% FBS. Endothelial cell line HUVEC was purchased from Life Technologies and maintained in HiEndoXL Endothelial Cell Expansion Medium (ECEM; HiMedia, Mumbai, India). HUVECs were used within three to five passages. miR-199a-3p and backbone vector overexpressing SNU449 cell lines were generated by G418 selection after transfecting SNU449 cells with premiR-199a-3p and vector plasmid, respectively. HepG2 cells were transfected using Lipofectamine 2000 (Life Technologies) and X-tremeGENE HP (Roche Diagnostics, Mannheim, Germany) was used for HUVEC, SNU449 and LX2 cells. In brief, 10^5^ of each cells were plated in 24-well Boyden chamber and transfected with 500 ng of each DNA following the manufacturer's instructions. If cells seeded on upper chamber were needed to transfect, we transfected separately and transferred to the transwell after 24–36 h. Authentication of cell lines was verified by short tandem repeat (STR) profiling.

### Plasmids used

pBABE-puro HGF was a gift from Bob Weinberg (Addgene plasmid #10901)^45^ and HGF-shRNA (clone number: TRCN0000003306) plasmid was purchased from Sigma-Aldrich (St. Louis, MO, USA). The plasmid expressing premiR-199a-3p was cloned in Dr. Thomas D Schmittgen's laboratory. Human VEGF recombinant protein was purchased from Life Technologies. Phospho-thiorate modified perfect complementary oligo [5′-TAACCAATGTGCAGACTACTGT-3′ modified in first two bases] was used to inhibit miR-199a-3p.

### Collection of liver tissues

HCC tumor and adjacent non-tumor tissues were obtained from patients who underwent orthotropic liver transplantation at the School of Digestive and Liver Diseases, Institute of Post Graduate Medical Education and Research (IPGME&R), Kolkata, India and the Center for Liver and Biliary Sciences, Indraprastha Apollo Hospital, New Delhi, India, with their prior written consent. Normal liver biopsies were collected from gall bladder carcinoma patients for routine evaluation of liver metastasis. The tissues were collected in RNA later (Ambion, Life Technologies, Carlsbad, CA, USA) and then preserved at −80 °C in a freezer. Ethics Committee of IPGME&R, Kolkata has approved the study.

### *In vivo* tumorigenicity and metastasis assay

All mice experiments were performed according to the Institutional Animal Care and Use Committee (IACUC) of the National Centre for Cell Science, Pune, India. miR-199a-3p or vector overexpressed SNU449 (1 × 10^6^) cells with matrigel (BD Biosciences, San Jose, CA, USA) at 1:1 dilution were injected subcutaneously into the right flank region of NOD/SCID mice (four mice in each group). The tumor volumes were measured twice a week using Vernier Caliper. After 4 weeks, the mice were killed and the tumor volumes were measured by using the formula *π*/6 ((*l* × *b*)^3/2^). For metastatic model, miR-199a-3p or vector overexpressed SNU449 (1 × 10^6^) cells were injected through lateral tail vein of NOD/SCID mice (four mice in each group), and after 4 weeks, the mice were killed and the number of nodules in the lung was counted and the lung tissue was stained with hematoxylin and eosin.

### Luciferase reporter assay

Binding aptitude of miR-199a-3p to the target gene was determined by luciferase-reporter assay using 3′-UTR of VEGFA, HGF, VEGFR1, VEGFR2, ANGPT1 and MMP2 cloned in psiCHECK2 vector (Promega, Madison, WI, USA) and transfected in HepG2 cells. Luciferase activity was determined using Dual-Luciferase Reporter Assay kit (Promega) following the manufacturer's protocol.

### Quantitative real-time PCR

Total RNA was isolated from different cell lines and liver tissues using TRIzol reagent following the manufacturer's instructions (Life Technologies). cDNA was synthesized using Superscript-III Reverse Transcriptase (Life Technologies) and quantified using SYBR Green PCR Master Mix (Roche Diagnostics) and gene-specific primers (Supplementary Table S2) in QuantStudio 7 Flex RealTime PCR system (Thermo Fisher Scientific, Waltham, MA, USA). MicroRNA was quantified using TaqMan miRNA Assay kit (Life Technologies). Data were normalized against 18 s rRNA or RNU6B.

### Immunoblot analysis and ELISA

Fifty micrograms of total cell lysate was separated on a 10% SDS-PAGE gel, transferred on PVDF membrane and blotted with antibodies. HGF, VEGFA, VEGFR2 and MMP2 antibodies were purchased from Santa Cruz Biotechnology (Dallas, TX, USA). ERK1/2, phospho-ERK1/2 and *α*-Tubulin were from Cell Signaling and Sigma, respectively. HRP-conjugated goat anti-rabbit and goat anti-mouse secondary antibodies were used to identify the protein of interest. Each experiment was repeated at least two times. Secreted VEGFA and HGF was detected either in conditioned media (CM) of cultured cells or patient's sera using ELISA kits from Ray Biotech (Norcross, GA, USA).

### Migration and invasion assay

*In vitro* migration assays were performed using Boyden chambers (BD Biosciences) with 8 *μ*m porous filters. LX2 cells were seeded in the lower and SNU449 cells in the upper compartment. After 24 h, migrated cells were counted from the lower surface of the membrane by staining with crystal violet. Average number of migrated cells from six different fields was presented in a bar diagram. For invasion assay, Matrigel basement membrane matrix coated transwell chambers (BD Biosciences) were used. SNU449 cells were seeded in the insert and cultured with CM of transfected LX2 cells diluted in 1:1 ratio with RPMI1640 media. Number of invaded cells in the lower chamber was determined as mentioned above.

### Tube-formation assay

HUVEC cells were transfected with premiR-199a-3p plasmid or vector. After 48 h, the cells were transferred on growth factor-reduced matrigel (Geltrex, Life Technologies)-coated 96-well culture plates and cultured for 8 h with either 20 ng/ml recombinant VEGF supplemented in ECEM or CM collected from different liver cell lines diluted with ECEM in 1:1 ratio. Photographs of cellular network formation were taken in a light microscope (EVOS, Life Technologies) and number of branches of the capillary network was counted to estimate *in vitro* angiogenesis. Mean of network branches of five visual fields were represented in bar diagram.

### Statistical analysis

Statistical analysis was performed in Microsoft EXCEL or GraphPad Prism software. All the data were presented as mean±standard deviation. Two tailed *t*-test and Mann–Whitney rank-sum test were used for data with normal and non-normal distribution, respectively.

## References

[bib1] Hanahan D, Weinberg RA. The hallmarks of cancer. Cell 2000; 100: 57–70. 10647931 10.1016/s0092-8674(00)81683-9

[bib2] Liotta LA, Kohn EC. The microenvironment of the tumour-host interface. Nature 2011; 411: 375–379. 10.1038/35077241 11357145

[bib3] Kalluri R, Zeisberg M. Fibroblasts in cancer. Nat Rev Cancer 2006; 6: 392–401. 16572188 10.1038/nrc1877

[bib4] Hernandez-Gea V, Toffanin S, Friedman SL, Llovet JM. Role of the microenvironment in the pathogenesis and treatment of hepatocellular carcinoma. Gastroenterology 2013; 144: 512–527. 23313965 10.1053/j.gastro.2013.01.002 PMC3578068

[bib5] Mbeunkui F, Johann DJJr. Cancer and the tumor microenvironment: a review of an essential relationship. Cancer Chemother Pharmacol 2009; 63: 571–582. 19083000 10.1007/s00280-008-0881-9 PMC2858592

[bib6] Witz IP, Levy-Nissenbaum O. The tumor microenvironment in the post-PAGET era. Cancer Lett 2006; 242: 1–10. 16413116 10.1016/j.canlet.2005.12.005

[bib7] Hanahan D, Coussens LM. Accessories to the crime: functions of cells recruited to the tumor microenvironment. Cancer Cell 2012; 21: 309–322. 22439926 10.1016/j.ccr.2012.02.022

[bib8] Wynn TA. Cellular and molecular mechanisms of fibrosis. J Pathol 2008; 214: 199–210. 18161745 10.1002/path.2277 PMC2693329

[bib9] Friedman SL, Roll FJ, Boyles J, Bissell DM. Hepatic lipocytes: the principal collagen-producing cells of normal rat liver. Proc Natl Acad Sci USA 1985; 82: 8681–8685. 3909149 10.1073/pnas.82.24.8681 PMC391500

[bib10] Nalesnik MA, Michalopoulos GK. Growth factor pathways in development and progression of hepatocellular carcinoma. Front Biosci (Schol Ed) 2012; 4: 1487–1515. 22652888 10.2741/s348

[bib11] Benjamin LE, Hemo I, Keshet E. A plasticity window for blood vessel remodelling is defined by pericyte coverage of the preformed endothelial network and is regulated by PDGF-B and VEGF. Development 1998; 125: 1591–1598. 9521897 10.1242/dev.125.9.1591

[bib12] Michiels C. Endothelial cell functions. J Cell Physiol 2003; 196: 430–443. 12891700 10.1002/jcp.10333

[bib13] Coulouarn C, Corlu A, Glaise D, Guénon I, Thorgeirsson SS, Clément B. Hepatocyte stellate cell cross-talk in the liver engenders a permissive inflammatory microenvironment that drives progression in hepatocellular carcinoma. Cancer Res 2012; 72: 2533–2542. 22419664 10.1158/0008-5472.CAN-11-3317 PMC3498759

[bib14] Yang JC, Teng CF, Wu HC, Tsai HW, Chuang HC, Tsai TF et al. Enhanced expression of vascular endothelial growth factor-A in ground glass hepatocytes and its implication in hepatitis B virus hepatocarcinogenesis. Hepatology 2009; 49: 1962–1971. 19475690 10.1002/hep.22889

[bib15] Deryugina EI, Quigley JP. Matrix metalloproteases and tumor metastasis. Cancer Metastasis Rev 2006; 25: 9–34. 16680569 10.1007/s10555-006-7886-9

[bib16] Carmeliet P, Jain RK. Molecular mechanisms and clinical applications of angiogenesis. Nature 2011; 473: 298–307. 21593862 10.1038/nature10144 PMC4049445

[bib17] Pillai RS. MicroRNA function: multiple mechanisms for a tiny RNA? RNA 2005; 11: 1753–1761. 16314451 10.1261/rna.2248605 PMC1370863

[bib18] Calin GA, Croce CM. MicroRNA signatures in human cancers. Nat Rev Cancer 2006; 6: 857–866. 17060945 10.1038/nrc1997

[bib19] Croce CM. Causes and consequences of microRNA dysregulation in cancer. Nat Rev Genet 2009; 10: 704–714. 19763153 10.1038/nrg2634 PMC3467096

[bib20] Tavazoie SF, Alarcón C, Oskarsson T, Padua D, Wang Q, Bos PD et al. Endogenous human microRNAs that suppress breast cancer metastasis. Nature 2008; 451: 147–152. 18185580 10.1038/nature06487 PMC2782491

[bib21] Png KJ, Halberg N, Yoshida M, Tavazoie SF. A microRNA regulation that mediates endothelial recruitment and metastasis by cancer cells. Nature 2011; 481: 190–194. 22170610 10.1038/nature10661

[bib22] Zheng F, Liao YJ, Cai MY, Liu TH, Chen SP, Wu PH et al. Systemic delivery of microRNA-101 potently inhibits hepatocellular carcinoma *in vivo* by repressing multiple targets. PLoS Genet 2015; 11: e1004873. 25693145 10.1371/journal.pgen.1004873 PMC4334495

[bib23] Kinose Y, Sawada K, Nakamura K, Sawada I, Toda A, Nakatsuka E et al. The hypoxia-related microRNA miR-199a-3p displays tumor suppressor functions in ovarian carcinoma. Oncotarget 2015; 6: 11342–11356. 25839163 10.18632/oncotarget.3604 PMC4484460

[bib24] Han Y, Kuang Y, Xue X, Guo X, Li P, Wang X et al. a novel target of miR-199a-3p, functions as a tumor suppressor in colorectal cancer. Biomed Pharmacother 2014; 68: 497–505. 24972723 10.1016/j.biopha.2014.05.003

[bib25] Huang J, Dong B, Zhang J, Kong W, Chen Y, Xue W et al. miR-199a-3p inhibits hepatocyte growth factor/c-Met signaling in renal cancer carcinoma. Tumour Biol 2014; 35: 5833–5843. 24609899 10.1007/s13277-014-1774-7

[bib26] Henry JC, Park JK, Jiang J, Kim JH, Nagorney DM, Roberts LR et al. miR-199a-3p targets CD44 and reduces proliferation of CD44 positive hepatocellular carcinoma cell lines. Biochem Biophys Res Commun 2010; 403: 120–125. 21055388 10.1016/j.bbrc.2010.10.130 PMC3039123

[bib27] Wu D, Huang HJ, He CN, Wang KY. MicroRNA-199a-3p regulates endometrial cancer cell proliferation by targeting mammalian target of rapamycin (mTOR). Int J Gynecol Cancer 2013; 23: 1191–1197. 23851675 10.1097/IGC.0b013e31829ea779

[bib28] Fornari F, Milazzo M, Chieco P, Negrini M, Calin GA, Grazi GL et al. MiR-199a-3p regulates mTOR and c-Met to influence the doxorubicin sensitivity of human hepatocarcinoma cells. Cancer Res 2010; 70: 5184–5193. 20501828 10.1158/0008-5472.CAN-10-0145

[bib29] Chen RX, Xia YH, Xue TC, Ye SL. Osteopontin promotes hepatocellular carcinoma invasion by up-regulating MMP-2 and uPA expression. Mol Biol Rep 2011; 520 38: 3671–3677. 10.1007/s11033-010-0481-8 21104439

[bib30] Gupta DK, Singh N, Sahu DK. TGF-β mediated 521 crosstalk between malignant hepatocyte and tumor microenvironment in hepatocellular carcinoma. Cancer Growth Metastasis 2014; 7: 1–8. 24741325 10.4137/CGM.S14205 PMC3988670

[bib31] Zhang Y, Yang P, Sun T, Li D, Xu X, Rui Y et al. miR-126 and miR-126* repress recruitment of mesenchymal stem cells and inflammatory monocytes to inhibit breast cancer metastasis. Nat Cell Biol 2013; 15: 284–294. 23396050 10.1038/ncb2690 PMC3672398

[bib32] Wang R, Zhao N, Li S, Fang JH, Chen MX, Yang J et al. MicroRNA-195 suppresses angiogenesis and metastasis of hepatocellular carcinoma by inhibiting the expression of VEGF, VAV2 and CDC42. Hepatology 2013; 58: 642–653. 23468064 10.1002/hep.26373

[bib33] Zhao Y, Adjei AA. Targeting angiogenesis in cancer therapy: moving beyond vascular endothelial growth factor. Oncologist 2015; 20: 660–673. 26001391 10.1634/theoncologist.2014-0465 PMC4571783

[bib34] Villaruz LC, Socinski MA. The role of anti-angiogenesis in non-small-cell lung cancer: an update. Curr Oncol Rep 2015; 17: 26. 25947099 10.1007/s11912-015-0448-y PMC4836185

[bib35] Koga K, Todaka T, Morioka M, Hamada J, Kai Y, Yano S et al. Expression of angiopoietin-2 in human glioma cells and its role for angiogenesis. Cancer Res 2001; 61: 6248–6254. 11507079

[bib36] Zhu K, Pan Q, Zhang X, Kong LQ, Fan J, Dai Z et al. MiR-146a enhances angiogenic activity of endothelial cells in hepatocellular carcinoma by promoting PDGFRA expression. Carcinogenesis 2013; 34: 2071–2079. 23671131 10.1093/carcin/bgt160

[bib37] Klein G, Vellenga E, Fraaije MW, Kamps WA, de Bont ES. The possible role of matrix metalloproteinase (MMP)-2 and MMP-9 in cancer, e.g. acute leukemia. Crit Rev Oncol Hematol 2004; 50: 87–100. 15157658 10.1016/j.critrevonc.2003.09.001

[bib38] Chen Q, Zhao X, Zhang H, Yuan H, Zhu M, Sun Q et al. MiR-130b suppresses prostate cancer metastasis through down-regulation of MMP2. Mol Carcinog 2014; 54: 1292–1300. 25154741 10.1002/mc.22204

[bib39] Monvoisin A, Neaud V, De Lédinghen V, Dubuisson L, Balabaud C, Bioulac-Sage P et al. Direct evidence that hepatocyte growth factor-induced invasion of hepatocellular carcinoma cells is mediated by urokinase. J Hepatol 1999; 30: 511–518. 10190737 10.1016/s0168-8278(99)80113-5

[bib40] Heideman DAM, Overmeer RM, van Beusechem VW, Lamers WH, Hakvoort TB, Snijders PJ et al. Inhibition of angiogenesis and HGF-cMET-elicited malignant processes in human hepatocellular carcinoma cells using adenoviral vector-mediated NK4 gene therapy. Cancer Gene Ther 2005; 12: 954–962. 15905856 10.1038/sj.cgt.7700856

[bib41] Wilhelm SM, Adnane L, Newell P, Villanueva A, Llovet JM, Lynch M. Preclinical overview of sorafenib, a multikinase inhibitor that targets both Raf and VEGF and PDGF receptor tyrosine kinase signaling. Mol Cancer Ther 2008; 7: 3129–3140. 18852116 10.1158/1535-7163.MCT-08-0013 PMC12261297

[bib42] Hong F, Chou H, Fiel MI, Friedman SL. Antifibrotic activity of sorafenib in experimental hepatic fibrosis: refinement of inhibitory targets, dosing, and window of efficacy *in vivo*. Dig Dis Sci 2013; 58: 257–264. 22918681 10.1007/s10620-012-2325-y PMC3543488

[bib43] Llovet JM, Ricci S, Mazzaferro V, Hilgard P, Gane E, Blanc JF et al. Sorafenib in advanced hepatocellular carcinoma. N Engl J Med 2008; 359: 378–390. 18650514 10.1056/NEJMoa0708857

[bib44] Cheng AL, Kang YK, Chen Z, Tsao CJ, Qin S, Kim JS et al. Efficacy and safety of sorafenib in patients in the Asia-Pacific region with advanced hepatocellular carcinoma: a phase III randomised, double-blind, placebo-controlled trial. Lancet Oncol 2009; 10: 25–34. 19095497 10.1016/S1470-2045(08)70285-7

[bib45] Gupta PB, Kuperwasser C, Brunet JP, Ramaswamy S, Kuo WL, Gray JW et al. The melanocyte differentiation program predisposes to metastasis after neoplastic transformation. Nat Genet 2005; 37: 1047–1054. 16142232 10.1038/ng1634 PMC1694635

