# Supplementary material for: MiRNA199a-3p suppresses tumor growth, migration, invasion and angiogenesis in hepatocellular carcinoma by targeting VEGFA, VEGFR1, VEGFR2, HGF and MMP2
Source: Cell Death Dis. 2017 Mar 30;8(3):e2706–. doi: 10.1038/cddis.2017.123 (PMC5386529; doi:10.1038/cddis.2017.123)

**Supplementary Data**

**Supplementary Table 1:** Nucleotide sequence alignments of miR-199a-3p to its target genes.

**Supplementary Table 2:** Sequences of primers for cDNA amplification of the genes studied.

**Supplementary Figure S1:** Expression of mature miR-199a-3p in SNU449 cells stably transfected with control vector and premiR-199a-3p.

**Supplementary Figure S2:** 3’UTR-reporter-luciferase assay of ANGPT1 in HepG2 cells transfected with control vector and premiR-199a-3p.

**Supplementary Figure S3: Expression of VEGFA, VEGFR1 and VEGFR2 in cell lines.** The mRNA expression of VEGFA, VEGFR1 and VEGFR2 were determined by semi-quantitative RT-PCR in different types of liver cell lines. SNU449 and HepG2 are hepatocellular carcinoma and hepatoblastoma cell lines respectively. HUVEC is human umbilical vein endothelial cells.

**Supplementary Figure S4: miR-199a-3p reduces intracellular HGF protein expression in LX2 cell line.** LX2 cells were transfected with vector, pBABE-puro HGF, HGF-shRNA and premiR-199a-3p. Intracellular HGF expression was determined by Immunofluorescence microscopy. Scale bar represents 20µm.

**
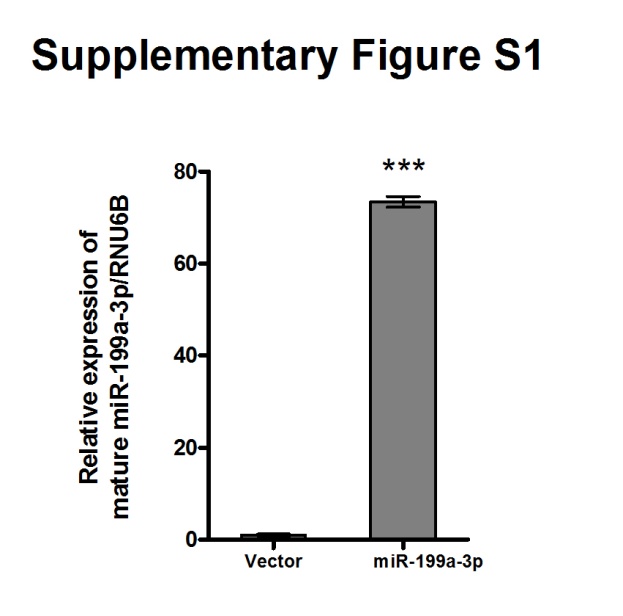
**


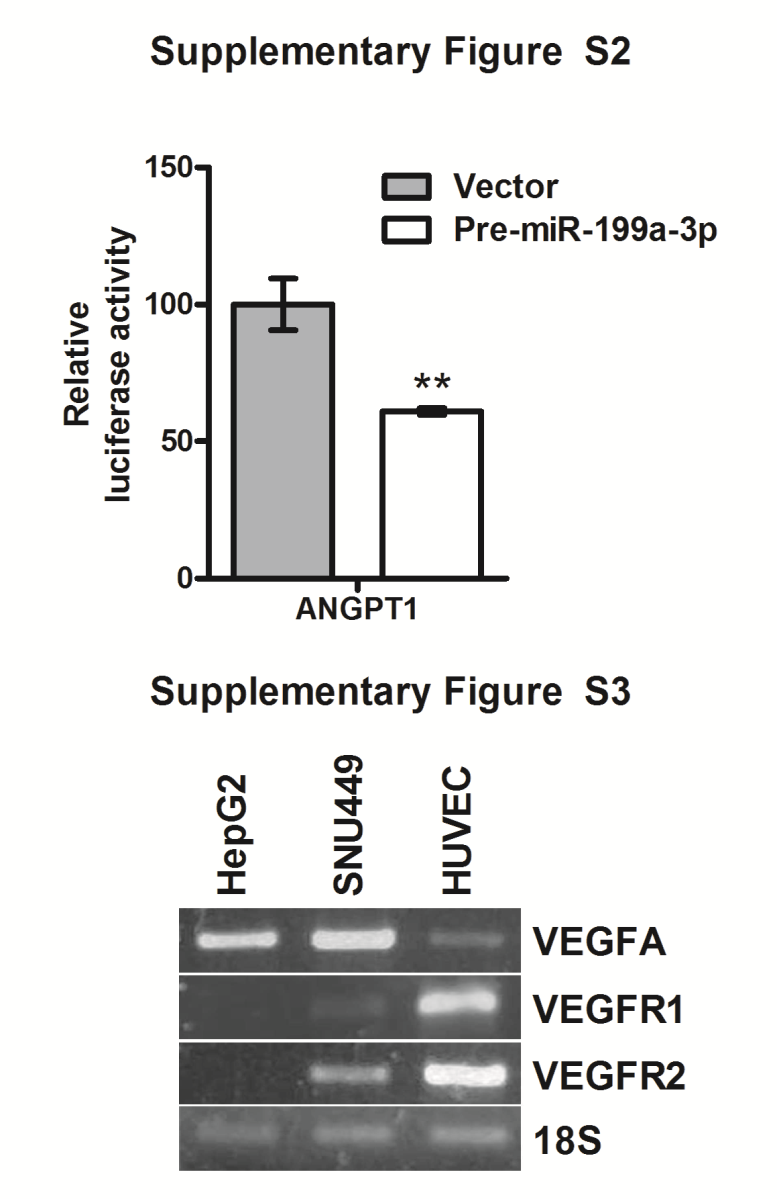


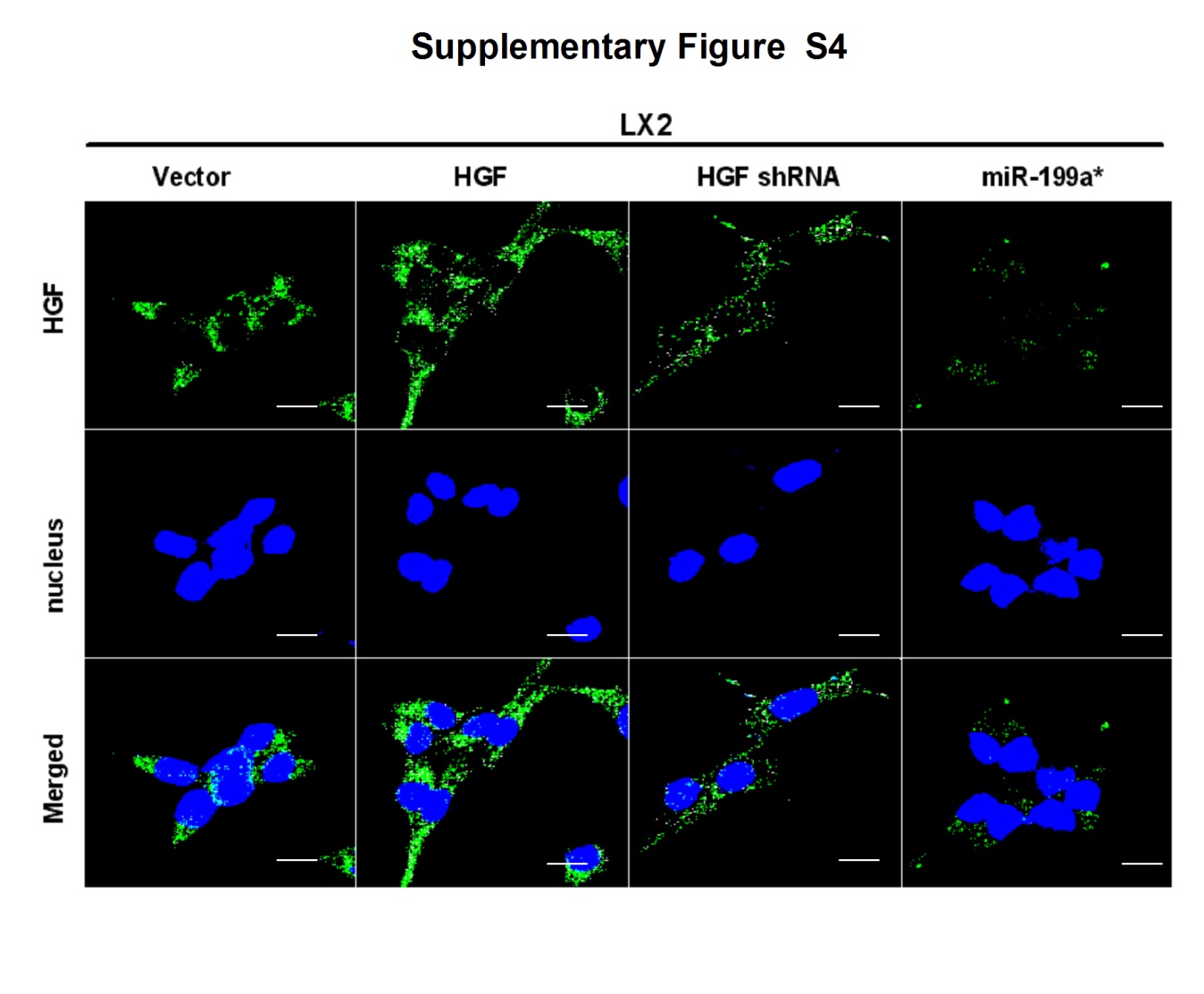

Supplement: Supplementary Information [file cddis2017123x1.docx]
